# Linked optical and gene expression profiling of single cells at high-throughput

**DOI:** 10.1186/s13059-020-01958-9

**Published:** 2020-02-24

**Authors:** Jesse Q. Zhang, Christian A. Siltanen, Leqian Liu, Kai-Chun Chang, Zev J. Gartner, Adam R. Abate

**Affiliations:** 1 Department of Bioengineering and Therapeutic Sciences, University of California San Francisco, San Francisco, CA USA; 2 UC Berkeley-UCSF Graduate Program in Bioengineering, University of California San Francisco, San Francisco, CA USA; 3 Department of Pharmaceutical Chemistry, University of California San Francisco, San Francisco, CA USA; 4 California Institute for Quantitative Biosciences, University of California San Francisco, San Francisco, CA USA; 5 Chan Zuckerberg Biohub, San Francisco, CA USA

**Keywords:** Index sorting, Single-cell RNA sequencing, Microfluidics, Flow cytometry

## Abstract

Single-cell RNA sequencing has emerged as a powerful tool for characterizing cells, but not all phenotypes of interest can be observed through changes in gene expression. Linking sequencing with optical analysis has provided insight into the molecular basis of cellular function, but current approaches have limited throughput. Here, we present a high-throughput platform for linked optical and gene expression profiling of single cells. We demonstrate accurate fluorescence and gene expression measurements on thousands of cells in a single experiment. We use the platform to characterize DNA and RNA changes through the cell cycle and correlate antibody fluorescence with gene expression. The platform’s ability to isolate rare cell subsets and perform multiple measurements, including fluorescence and sequencing-based analysis, holds potential for scalable multi-modal single-cell analysis.

## Introduction

Cellular processes, such as replication, migration, and differentiation, are tightly controlled by signaling and gene regulatory networks [1–3]. These processes are dynamic, and at any point, a cell may exist along a continuum of states [4]. Thus, cell state heterogeneity is often masked when bulk methods are used to analyze populations [5, 6]. The development of high-throughput single-cell RNA sequencing (scRNA-seq) has enabled populations to be analyzed at the single cell level [7–9], facilitating the dissection of cellular heterogeneity and the construction of an atlas of cell states across the human body [10]. However, gene expression is just one dimension by which cells may be characterized, and many properties, such as epigenetic state, protein expression, enzyme activity, and cellular morphology, are not readily measured by scRNA-seq [11, 12].

More comprehensive cell characterization can be accomplished by combining scRNA-seq with complimentary measurement modalities. Optical approaches, including microscopy and flow cytometry, can characterize morphological and fluorescence phenotypes prior to scRNA-seq [13, 14]. Linked optical analysis and scRNA-seq has been applied to in vitro cultures, patient tissues, and stem cells, revealing molecular links to cellular function [15–17]. While powerful, these approaches are limited in throughput [15, 18]. Cytometry methods are more scalable since the instrument can automatically sort cells into wells for automated library preparation, increasing throughput to hundreds of cells [16, 17, 19]. Going beyond this, however, is impractical because the time and volume of reagent required to process tens or hundreds of thousands of cells is prohibitive [20]. Recent spatial transcriptome sequencing approaches might ultimately enable scalable imaging and scRNA-seq but rely on methods to image and label cells that are not yet standard in the field [21, 22].

Microwell technologies improve throughput and can process thousands of single cells for RNA sequencing with the benefit of allowing cells to be imaged prior to sequencing preparation [23, 24]. However, because these platforms load cells stochastically, most wells remain empty, limiting the total number of cells analyzed to ~ 2000. Moreover, there is no integrated enrichment in these methods, making it challenging to isolate rare cell subsets, which are important in a variety of applications, including cancer pathophysiology, immunology, and stem cell biology [25–27]. For example, for a target cell present at 5%, only ~ 100 cells would be captured, yielding a throughput no better than flow cytometric methods. To enable optical and sequence-based analysis of specific cells in heterogeneous samples, a new approach is needed that can specifically isolate, perform optical measurements on, and sequence large numbers of target cells.

In this paper, we present high-throughput optical and RNA sequencing analysis of single cells. Our instrument functions like a flow cytometer, optically scanning cells in flow and sorting targets into individual nanoliter wells for sequencing preparation. To pair the optical and sequencing data, the wells are indexed with coordinate oligos that are captured during sequencing; this allows all reads for a given cell to be associated with a specific well on the plate, thereby pairing it with the optical data. The cell analysis is accomplished at ~ 1 kHz and dispensing at ~ 5 Hz, allowing hundreds of rare cells to be isolated in a few minutes. The total volume of reagent on the array is ~ 1 μL per 1000 single-cell transcriptomes, representing a 1000-fold reduction compared to conventional microliter well plates. Moreover, with standard fabrication techniques, ~ 10,000 wells can be fabricated on a microscope slide, providing a scalable means by which to acquire linked single cell optical and gene expression data for selected cell populations.

## Results and discussion

**Fig. 1:**
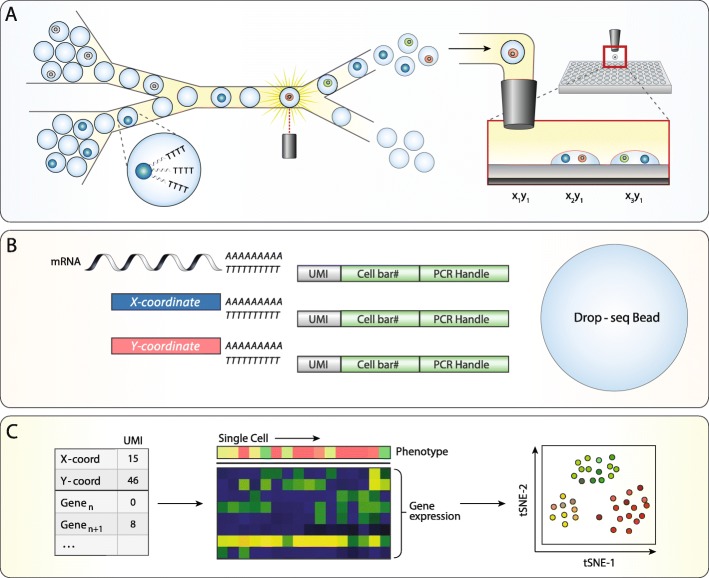
A high-throughput platform for linked optical phenotype and gene expression of single cells. **a** Monodisperse droplet emulsions containing encapsulated poly-T mRNA capture beads and cells are input into a microfluidic device. Fluorescence signal from droplets is interrogated and used to selectively dispense a cell and bead to indexed locations on a nanowell array. **b** Each bead binds mRNA from cell lysate as well as a unique combination of poly-A barcode oligos denoted by nanowell coordinate. **c** UMI counts on each bead are collected through sequencing into an expression matrix for each cell. Nanowell coordinate is assigned based on the abundance of barcode oligos and paired with fluorescence data obtained during cell sorting, which enables downstream linked analyses such as dimensionality reduction visualizations of gene expression paired with optical phenotype

Our single-cell analysis platform is based on Printed Droplet Microfluidics (PDM) [28, 29], an approach that allows cells to be optically scanned and dispensed to custom nanoliter well plates (nanoplates) (Fig. 1a). To perform linked optical and scRNA-seq analysis, we record the fluorescence of a cell while confined to a droplet, then dispense the cell and droplet to the nanoplate at defined locations. Then, scRNA-seq library preparation is performed on each cell using specific “coordinate oligos” encoding each cell’s location on the nanoplate. After sequencing, these oligos allow each cell barcode to be traced to a well of origin, thereby linking it to the optical data collected for that cell. The workflow is similar to flow cytometry, except that the sorter is a microfluidic device and the wells in which the cells are dispensed are ~ 10,000-fold smaller than conventional microwells. This reduction in volume, combined with the speed of the microfluidic printer, enables highly scalable optical phenotyping and sequencing of single cells.

Prior to device operation, a separate flow focusing device encapsulates cells in a droplet emulsion. We introduce this emulsion into the PDM device, where each drop is optically scanned (Fig. 1a, left). As in flow cytometry, laser-induced fluorescence accomplishes the optical analysis, whereby focused lasers excite fluorescence of the cells which a multicolor detector then captures (Fig. 1a, middle). Cells with desired fluorescence properties are isolated through sorting them into a printing nozzle that dispenses them into a nanowell on the substrate (Fig. 1a, right). We record the cell fluorescence and dispense location, allowing this information to be paired with the scRNA-seq data collected later.

To link the optical and sequencing data, we index the wells such that each cells’ dataset can be traced back to a well on the array. The indexes comprise “coordinate oligos” pre-loaded into the wells using a commercial reagent spotter (Fig. 1b, lower left) [30]. To index the array, we place “X” and “Y” coordinate oligos, each of which contains a different 8 base sequence encoding the specific location of a given nanowell on the plate. The coordinate oligos are polyadenylated, allowing them to be captured along with cellular mRNA during the scRNA-seq library preparation. For scRNA-seq, we adapt the validated “Drop-Seq” protocol [7], which uses beads coated with poly-deoxythymidine “barcode” oligos to capture and label both mRNA and coordinate oligos. We accomplish this by co-dispensing beads and cells in each nanowell and lysing the cells. After retrieving the beads, performing the requisite library preparation steps of Drop-Seq, and sequencing the barcoded cDNA, we obtain a collection of reads representing the cell transcriptome and coordinate oligos, all sharing a Drop-Seq barcode. Thus, the location of the cell from which the data originate is encoded in the sequencing data, allowing it to be traced back to a specific well on the array (Fig. 1c, left) and associated with the previously recorded optical data (Fig. 1c, middle). With this paired dataset, we can use dimensionality reduction methods to first visualize gene expression data, to which we add the optical phenotype information (Fig. 1c, right).

**Fig. 2:**
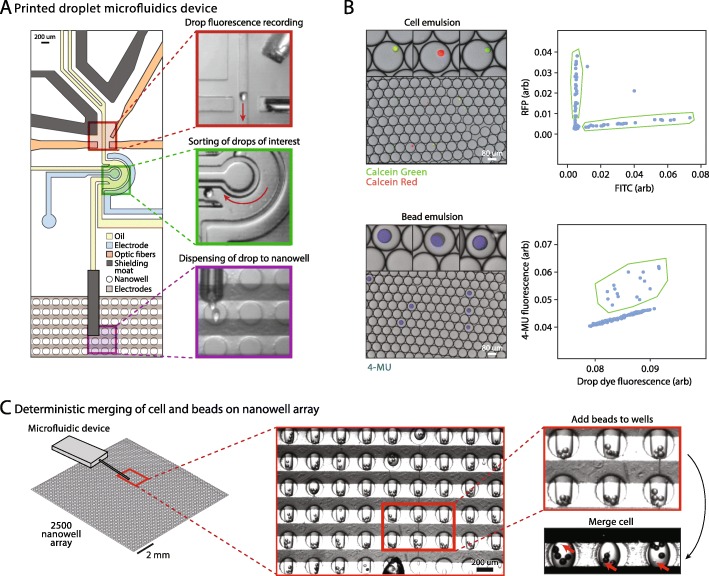
Printed Droplet Microfluidics (PDM) operation for deterministic loading of nanowell array with beads and cells. **a** An inset of the microfluidic device aligned over the nanowell array, with images (top to bottom) of regions of drop fluorescence recording, sorting of drops of interest, and dispensing of drops to nanowells. **b** Monodisperse droplet emulsions containing fluorescently labeled cells (top) or beads (bottom) are input into PDM. Drops of interest (insets) are enriched for by gating on fluorescence plots (right) generated during device operation. **c** Deterministic merging of cells and beads through first adding beads to nanowells, followed by merging of a cell-containing drop in lysis buffer

The microfluidic print device consists of a droplet spacer, sorter, and printing nozzle (Fig. 2a). A packed emulsion containing cells or beads is introduced, spaced by oil, and optically scanned by a four-color laser-induced fluorescence detector (Fig. 2a, red outline). Embedded fiber optics excite and collect fluorescence that is processed through filters and analyzed in real time by custom software; this allows cell, bead, and droplet fluorescence and scattering to be recorded, to determine whether to print the droplet and its contents to the current nanowell. Printing is achieved by sorting a droplet (Fig. 2a, green outline) into the printing nozzle positioned above a nanowell (Fig. 2a, purple outline); if the current droplet should not be printed, it is not sorted into the nozzle and passes into the discard channel. Because the carrier oil is viscous and denser than water, in the absence of other forces, the ejected droplet would float away and not go into the nanowell. Thus, to dispense it into the nanowell, electrodes positioned under the substrate emit an oscillating electric field. This field pulls the dispensed droplet into the nanowell by a dielectrophoretic force and is key to the speed of PDM, since it allows a droplet to travel the final few hundred microns from the printing nozzle to the nanowell in tens of milliseconds [28]. Moreover, because the trap extends above the substrate, the printer need not dispense the droplets with perfect accuracy into the nanowells, since any droplet within the electric field will, ultimately, be pulled into the nearest nanowell. The trapping field also ensures that the printed droplets remain fixed in the wells. Upon completion of a print run, droplets can be released by un-powering the electrodes (Additional file 1: Movie S1).

To demonstrate the accuracy of scRNA-seq using our approach, we perform an experiment with two cell types. We prepare and encapsulate a mixed suspension of Calcein Red stained mouse (3T3) and Calcein Green stained human (HEK293) cells (Fig. 2b, upper left). When scanned in the print head, we observe distinct green and red cell populations (Fig. 2b, upper right); thus, with suitable gating instructions, the printer can print these cells in a defined pattern to the nanoplate. To enable scRNA-seq of the printed cells, Drop-Seq beads must also be printed, which requires that they be detectable in the print head; this is accomplished by labeling them with 4-MU, a blue dye that does not overlap with the cell stains (Fig. 2b, lower left). The 4-MU dye is insoluble in water and remains sequestered within the beads following labeling, allowing bead-loaded to be discerned from bead-empty droplets (Fig. 2b, lower right). To print cells and beads in defined combinations, we generate a “print file” containing gating and location instructions that we input into the printer software; the printer reads this file, printing cells and beads to the nanoplate according to the instructions in the file (Fig. 2c, left).

**Fig. 3:**
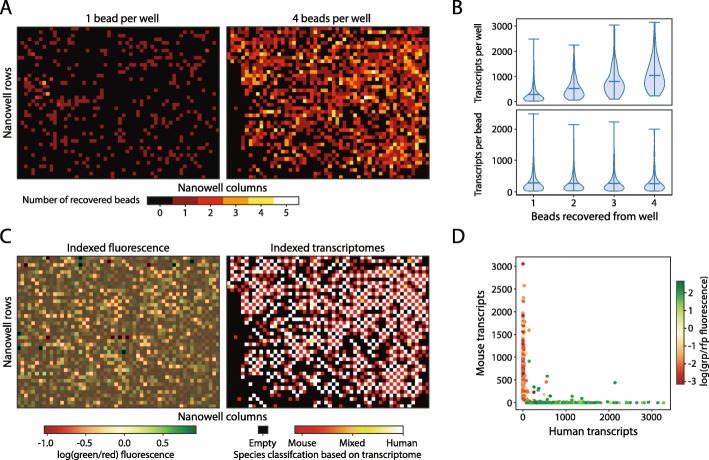
Linked optical phenotype and gene expression measurements verified with two species experiment. **a** One or 4 beads were printed to each well of a 42 by 56 nanowell array along with an alternating pattern of mouse and human cells. The number of recovered beads per nanowell position was determined by the number of unique cell barcodes mapped back to each nanowell. **b** When printing four beads per well, the distribution of transcripts recovered from each nanowell was calculated as a function of the number of beads recovered. The distribution of the number of transcripts originating from each bead within a nanowell was also plotted as a function of the number of beads recovered per nanowell. **c** Left: fluorescence data from alternating printing of Calcein Green stained human cells and Calcein Red stained mouse cells indexed by nanowell position. Right: ratio of human to mouse transcripts recovered from each nanowell based on printing four beads per nanowell. **d** Transcript counts by nanowell position are annotated with the green-red fluorescence ratio from the cell printed into the corresponding nanowell

A strength of PDM for scRNA-seq is that it allows the nanowells to be controllably loaded with cells and beads; this contrasts with other high-throughput scRNA-seq methods which randomly load cells or beads and, thus, are less efficient. Moreover, PDM allows systematic variation of nanowell contents across the array, to choose conditions that maximize data quality [28]. For instance, minimizing the rate of doublets—when two cells are inadvertently sequenced as one—leads to better data quality and lower per-cell sequencing costs [31, 32]. While we cannot directly monitor the number of cells passing through each droplet, in the two-cell experiment, we gate out heterotypic doublets of droplets containing a red and green cell and minimize the rate of homotypic doublets by excluding the upper tail of the Calcein Red and Calcein Green distributions. Moreover, controlled printing allows us to load multiple capture beads to every well (Fig. 2c, right, Additional file 2: Movie S2), which can increase cell and mRNA capture efficiency by compensating for losses during sequencing library preparation [33]. To illustrate this, we print two substrates, the first with 1 bead per well and the second with 4, both on 42 by 56 nanowell (2352) arrays. All beads originating from the same well contain the same coordinate barcodes, allowing us to group reads associated with multiple beads together. Due to loss of beads during library preparation, starting with more beads per well increases the likelihood of recovering at least one bead from every well (Fig. 3a). When printing more beads, we also recover more transcripts per well (Fig. 3b, upper), and that the number of transcripts per bead remains consistent when recovering up to four beads per well (Fig. 3b, lower). This suggests that increasing bead surface area per cell lysate increases mRNA capture.

Single-cell RNA-seq methods depend on minimal cross contamination of RNA between cells [31]. To investigate cross contamination, we print mouse and human cells in a checkerboard pattern and classify each transcriptome according to which species’ genome the transcripts predominately align. We find that the optical data align 99.3% of the time with the expected printing pattern (Fig. 3c, left). We recover transcripts from 1204 nanowells following sequencing and bioinformatic analysis, with 97.6% of transcriptomes having species purities of at least 90% mouse or 90% human and matching the expected printing pattern (Fig. 3c, right). As we demonstrate a near-perfect success rate when printing beads and cells, we reason that the most of the lost nanowells represent beads that were not collected after printing or lost during library preparation. We find that 1.8% of nanowells have less than 90% species purity, suggesting either mRNA cross contamination or misprinting of cells (dots not aligned with axes) (Fig. 3d). When we sequence deeper on a smaller subset of beads, we recover on average 17,500 and 15,200 transcripts from mouse and human cells, respectively (Additional file 3: Fig. S1). In sum, we demonstrate quality optical and scRNA-seq data from over a thousand cells when printing four beads per well.

**Fig. 4:**
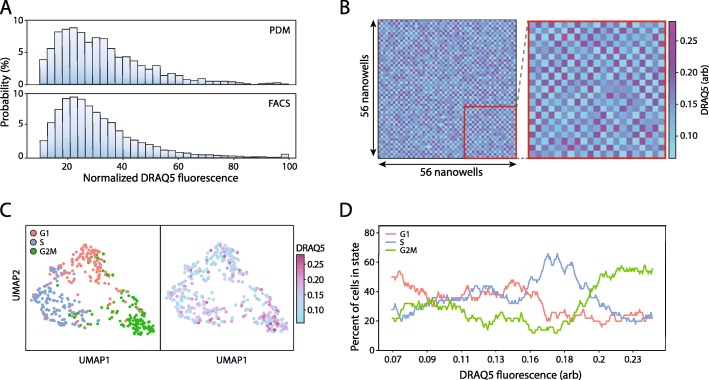
Linked fluorescence and gene expression analysis of cell cycle state in Jurkat cells stained with a DNA-binding dye. **a** The frequency distribution of Jurkat cells stained with DRAQ5 encapsulated within droplets was analyzed on both PDM and a flow cytometer. **b** An alternating pattern of high and low expressing DRAQ5 Jurkats was dispensed to a 56 by 56 nanowell array using PDM. Fluorescence measurements were indexed by nanowell position (inset). **c** Transcriptomes from 498 cells were recovered and clustered based on cell cycle state (left) predicted based on a set of cell cycle-dependent genes. DRAQ5 fluorescence data collected during printing was then overlaid (right). **d** Cells were ordered by low to high DRAQ5 signal, and the fraction of cells in each cell cycle state was calculated over a 50-cell sliding window using corresponding cell cycle state assignments by gene expression analysis

Cells undergo changes in state and phenotype through the cell cycle [17]. For example, by the G2 phase, cells have doubled their genome, allowing it to be optically detected via DNA staining [34]. Moreover, different genes peak and diminish in expression through the cycle, making the cell cycle useful for validating our approach. As a model system, we use Jurkat cells stained with DRAQ5, which is a live-cell stain for genomic DNA (Fig. 4a, upper). We observe a broad distribution of DNA fluorescence, the brightest of which likely correspond to cells with the most DNA and, thus, in the G2 or M phase of the cell cycle. To confirm these results, we perform flow cytometry of the suspension and obtain a similar distribution (Fig. 4a, lower). We utilize PDM to generate a 56 by 56 nanowell array to which we print a checkerboard pattern of low and high DRAQ5 expressing cells (Fig. 4b). We sequence transcriptomes from 437 cells and use Uniform Manifold Approximation and Projection (UMAP) to visualize these cells [35]. Through assigning cells to G1, S, or G2M phases based on their expression of cell cycle-associated genes and using those genes for principal component analysis, we generate a UMAP plot which identifies three clusters in agreement with three stages of the cell cycle (Fig. 4c, left). To determine whether these classifications agree with the optical data, we annotate the points of the UMAP plot according to the magnitude of DRAQ5 fluorescence (Fig. 4c, right). The plots are in general agreement, with the state comprising the most DNA (G2M) appearing brightest in the DNA stain. To observe how the population varies through this cycle, we order the cells by fluorescence and plot the proportion in the three states as classified by gene expression (Fig. 4d). We expect the proportion of cells in G1 to be at low DRAQ5 signal, S phase in the middle of the distribution, and G2 at the top end. The peak of the G1 phase curve is at the low end of the DRAQ5 distribution, S phase in the middle, and G2/M at the top end. We observe general concordance with the expected trend when we pair fluorescence and scRNA-seq measurements of cell cycle state. With our platform, we thus demonstrate characterization of a fundamental biological process through linked optical and gene expression analysis.

**Fig. 5:**
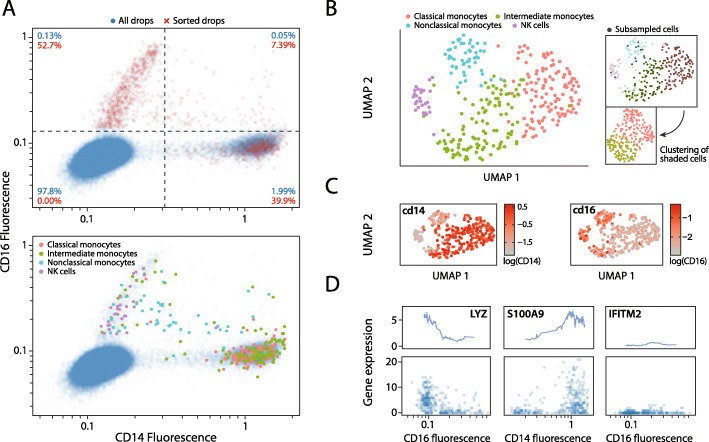
Paired optical phenotype and gene expression analysis of enriched CD14+/CD16+ cells from PBMCs. **a** Use of dual antibody panel and PDM to significantly enrich for CD14+ and CD16+ cells from PBMCs encapsulated within droplets (top). Percentages represent quadrant proportions, with red indicating post-sort frequency. Annotation of sorted cells by cell type as identified through RNA-seq (bottom). **b** UMAP of bioinformatically filtered cells clustered by cell type (left). Cells are subsampled such that the ratio of CD14+ to CD16+ matches the observed pre-sorting ratio; those cells are clustered (right). **c** Overlays of CD14 and CD16 fluorescence for each cell on the UMAP. **d** Scatter plots of gene expression and fluorescence data for 3 selected genes, with moving average of expression plotted above (bin size = 30)

Cell populations are often heterogeneous, with particularly important cells present below 5%, such as those involved in cancer pathogenesis, immunological memory, and maintaining adult stem cell niches [25–27]. With PDM, we can enrich for rare cell types using their optical phenotype prior to their loading on the nanowell array, markedly increasing the abundance of rare cell types in the obtained sequencing data. We demonstrate this capability by enriching for CD14-positive (CD14+) and CD16-positive (CD16+) peripheral blood mononuclear cells (PBMCs). We count CD14+ and CD16+ cell abundances prior to analysis using PDM and determine that they constitute ~ 25% and ~ 3% of all PBMCs, respectively. We load the nanowell array with CD14+ and CD16+ PBMCs, demonstrating an up to 405-fold enrichment of cells over their detection frequencies (Fig. 5a, upper). We recover transcriptomes from 310 cells and determine their phenotype through gene expression analysis. By overlaying the cell phenotype over the fluorescence data, we determine that natural killer (NK) cells largely are CD14−/CD16+, nonclassical monocytes CD14+/CD16+, and classical monocytes and monocytes of an intermediate phenotype CD14+/CD16− (Fig. 5a, lower). These four phenotypes cluster separately on a UMAP (Fig. 5b, left). By contrast, when we sample cells from the sequencing data such that the ratios of CD14+ to CD16+ cells match the observed abundances, we do not resolve the NK or nonclassical monocyte clusters after clustering (Fig. 5b, right). The ability of our approach to enrich for specific cells in a heterogeneous population and dedicate all the sequencing to them thus provides a powerful advantage when the cells of interest are rare.

Flow cytometry is routinely used to profile monocyte phenotypes based on CD14 and CD16 surface marker expression [36]. By overlaying the optical data on the UMAP, we show that CD14+/CD16− cells largely correlate with the classical and intermediate monocyte clusters, and the NK cells and nonclassical monocyte clusters are CD16+ (Fig. 5c). We also observe an intermediate monocyte population that is CD14+/CD16+. To further study the trends between optical phenotype and gene expression, we plot gene expression versus CD14 or CD16 fluorescence for three relevant genes: LYZ, S100A9, and IFITM2 (Fig. 5d). LYZ and S100A9 are associated with the classical monocyte phenotype [37], and IFITM2 is associated with nonclassical monocytes [38]. By ordering cells from low to high fluorescence and taking the moving average of gene expression, we uncover trends between fluorescence and gene expression for these genes. LYZ expression decreases with increasing CD16 fluorescence; S100A9 expression increases with increasing CD14 fluorescence; IFITM2 expression is globally lower than the others, but expression peaks in the middle of the CD16 fluorescence distribution. By combining fluorescence and gene expression measurements, we highlight that the expression of signature monocyte phenotype-associated genes gradually changes with optical phenotype.

Single-cell RNA-seq is a powerful and general method for analyzing cells, but not all traits of interest are observable in gene expression data alone. Here, we demonstrate an approach that allows gene expression to be linked to optical data in a high-throughput format scalable to thousands of single cells. A key concern for any scRNA-seq workflow that relies on barcoding cells for bulk sample amplification is the loss of cells due to loss of beads during downstream processing [33]. By spreading each cell’s transcriptome over several beads, we increase the probability of recovering transcriptomes for all cells. Our platform’s ability to localize combinations of cells, beads, and reagents at defined positions on a nanoliter array affords other powerful capabilities, such as systematic variation of cell, reagent, and drug combinations and tuning of optical and sequencing parameters to achieve optimal data. The open nature of the array also makes it amenable to additional measurement modalities, such as atomic force microscopy, mass spectrometry, and chemical assays, all of which can be linked with optical and scRNA-seq data using the approach we have presented [39, 40]. The ability to link sequencing readouts with other measurements for thousands of single cells will facilitate further investigations into the molecular underpinnings of cell function [41].

## Materials and methods

### Microfluidic device fabrication

PDM chips are fabricated by poly(dimethylsiloxane) (PDMS) molding over a SU-8 master. Briefly, a three-layer SU-8 negative master is patterned to form 20, 80, and 150 μm tall features using previously described multi-layer SU-8 photolithography techniques [28]. Following casting of PDMS over the SU-8 and curing at 65° for 2 h, inlet holes are punched into devices using a 0.75-mm biopsy core. Devices are then plasma bonded to 25 mm × 75 mm glass slides. One centimeter of PE/5 tubing (Scientific Commodities) is inserted into the nozzle channel and sealed with a 1-min instant mix epoxy (Norland). Channels are then treated with AquaPel (AquaPel). Drop-making devices are fabricated as previously described [28]. Two devices with a T-junction cross-section of 80 μm × 45 μm and 80 μm × 80 μm are used.

### Nanoplate fabrication

A negative of the electrode pattern is fabricated on a 50 mm × 75 mm glass slide by positive resist photolithography. A 2-μm-thick layer of MA-P 1215 (Micro Resist Technology) is spin-coated onto the slide and baked for 1 min on a 95° hotplate. The slide is then exposed to collimated 190 mW UV light (Thorlabs) for 3.5 min. The slide is developed in MF-24A developer (Dow Chemical) for 1 min. Patterned slides then have a 200-A-thick layer of chromium deposited on them (LGA Thin Films). The removal of the photoresist with acetone yields the electrode pattern. Nanowells are fabricated on electrode slide by first masking off the regions of electrode contact and spin-coating a 15-μm-thick layer of uncured PDMS. PDMS is then cured for 3 min on a 95° hotplate. Following plasma treatment of the slide, a 40-μm-thick layer of SU-8 is spin-coated onto the slide and allowed to soft-bake on 95° for 10 min. The slide is exposed to UV light under a photomask for 90 s, followed by 5 min of post-exposure baking at 95°. The slide is then immersed in PGMEA developer (Sigma) for 5 min, rinsed with PGMEA and isopropanol, and then dried on the hot plate for 2 min. Slides are then plasma treated and placed in a petri dish adjacent to reservoirs of trichloro(1H,1H,2H,2H-perfluorooctyl)silane (Sigma) for 2 h under vacuum at room temperature.

### Nanowell coordinate indexing

Nanowells are barcoded using the sciFLEXARRAYER S3 (Scienion AG). A 96-well “source-plate” containing up to 44 coordinate oligos (Additional file 3: Table S1, Table S2) diluted to 1 nM in DI water is prepared. Two nanoliters of each barcode oligo solution is dispensed to nanowells according to a pre-programmed print routine to label each nanowell with a unique but known combination of three oligos (Additional file 3: Fig. S2). Nanowells are split into 14-by-14 subarrays, of which each subarray had 14 unique *x* and 14 *y* coordinate oligos. Subarrays are tiled together, with each subarray having a unique *z* coordinate oligo, until the array reached the desired size. Following printing, slides are placed in a petri dish and sealed with parafilm and stored at − 20° until ready to use.

### PDM operation and optical configuration

A multimode excitation fiber with a core diameter of 105 μm and a NA of 0.22 (Thorlabs) is inserted into a guide channel in the PDM device. Similarly, an emission detection fiber with core diameter of 200 μm and NA of 0.39 (Thorlabs) is inserted into a second guide channel in the PDM device. Four 50 mW continuous wave lasers with wavelengths of 405, 473, 532, and 640 nm are combined and coupled to the excitation fiber. Emitted light is columnated and ported into a quad-bandpass filter, then passed through a series of dichroic mirrors. Bandpass filters of 448, 510, 571, and 697 nm past each dichroic mirror enable wavelength-specific detection of emitted light by PMTs. Electrode channels and a “Faraday moat” are filled with a 5 M NaCl solution. A positive electrode is connected to a function generator and a high voltage amplifier while a second electrode is grounded. Fluidic inputs into the PDM device are driven by syringe pumps (New Era). Bias and spacer oil containing 0.2% w/w IK in HFE-7500 are flowed through the device at a flow rate of 2000 μL/h. A waste channel is driven with a negative flow rate of − 3000 μL/h. Monodisperse droplet emulsions are reinjected into the device at a flow rate of 100 ± 50 μL/h. Real-time optical signal acquisition through a field programmable gate array (National Instruments) is displayed on a LabView software. Optical signal is processed in real time and displayed on a fluorescence dot plot, in which drop types of interest can be assigned by specifying gates. Droplets are subsequently sorted by passing a high frequency pulse through a high voltage amplifier (Trek 690E-6). Typical droplet sorting parameters range from 10 to 20 kHz, 50 to 100 cycles, and 0.5 to 1.0 kV. Copper tape with a conductive adhesive (Ted Pella) is affixed to two electrode contact pads on the nanoplate. One pad is connected to ground, while the other one is connected to a function generator and a high voltage amplifier, providing power at 200–600 V at 20–30 kHz. Slides are immersed in a bath of 2% w/w IK in FC-70 (3 M) during printing operation.

### Cell culture

HEK and 3T3 cells (ATCC) are cultured in 75 cm^2^ flasks in the presence of Dulbecco’s modified Eagle’s medium (DMEM) supplemented with 10% fetal bovine serum (FBS) and 1× Penicillin-Streptomycin at 37° and 5% CO_2_. Cells are treated with 0.25% Trypsin-EDTA and washed with media to generate cell suspensions. The viability and cell concentration are counted by a TC20 automated cell counter (BioRad). Cell suspensions are diluted to 1 million/mL in media. Suspensions are pelleted at 400 g for 3 min and resuspended in 1 mL DPBS. The HEK suspension is treated with 1 μg/mL of Calcein Green (Thermo-Fisher) while the 3T3 suspension is treated with 2 μg/mL of Calcein Red (Thermo-Fisher) for 15 min at 37°, followed by the addition of 4 mL media. Suspensions are pelleted and resuspended in media. Cells are mixed together in a 1:1 ratio and diluted in DPBS to form a final concentration of 250k/mL, which contained also 10 μM Cascade Blue-Dextran (Thermo-Fisher) and 0.5 v/v% FBS are added. Jurkat cells (ATCC) are cultured in RPMI-1640 medium supplemented with 10% FBS and 1× Penicillin-Streptomycin at 37° and 5% CO_2_. One million cells are extracted and pelleted at 400 g for 3 min and diluted in 500 μL DPBS, to which 1 μL of 5 mM DRAQ5 (Thermo-Fisher) is added. Cells are incubated at 37° for 5 min, to which 500 μL of DPBS is added which also contained 10 μM Cascade Blue-Dextran and 0.5 v/v% FBS.

### PBMC staining

Fresh-frozen human PBMCs are obtained from Stemcell Technologies. DMEM with 10% FBS is warmed up to 37°, and the frozen PBMCs are thawed by adding 1 mL of warm media on top of the frozen cells and immediately transferring the media to a 15-mL conical. This process is repeated several times, and then, warm media is added until the total volume in the conical was 10 mL. Cells are pelleted at 300 g for 5 min, after which the supernatant is removed and replaced with 10 mL of warm media. Cells are counted, and 500k cells are transferred into a 1.5-mL tube. After pelleting, the supernatant is removed and replaced with 1 mL ice-cold 1% BSA in PBS. Cells are pelleted, the supernatant is removed and replaced with 100 μL 1% BSA in PBS, and the tube contents are transferred to a 15-mL conical. Five microliters of FcX blocking antibody (BioLegend) is added, and the tube was incubated at room temperature for 5 min. Then, 7.5 μL of PE-conjugated CD16 antibody (BioLegend) and PE-Cy5.5-conjugated CD14 antibody (BioLegend) is added to the tube. Cells are stained at room temperature in the dark for 30 min. Cells are pelleted and washed twice with 5 mL 0.04% BSA in PBS, after which cells are resuspended in 1 mL 0.04% BSA in PBS, to which 2 μL of 1 mM FITC and 200 U RNAse inhibitor (Lucigen) is added. Cells are passed through a 40-μm cell strainer (Fisher Scientific) prior to encapsulation in droplets.

### Cell and bead encapsulation within monodisperse droplet emulsions

Barcoded mRNA capture beads are purchased through ChemGenes (MACOSKO-2011-10) and have a structure previously reported [7]. Beads arrived as a dry resin and are resuspended, washed, and filtered as previously described. For each experiment, 100,000 beads are extracted from the suspension and pelleted by placing on a tabletop centrifuge for 10 s. The supernatant is removed and replaced with 40 μL of 10 mM 4-MU (Sigma) in methanol diluted in 960 μL DPBS. The pellet is resuspended and allowed to stain for 1 min at room temperature. Beads are then pelleted, washed with DPBS once, and then resuspended in a solution of 10 μM FITC in DPBS to which is added 500 μL of the Drop-Seq lysis buffer. Beads are then placed into a 3-mL syringe with a magnetic stir bar (V&P Scientific) and encapsulated in 2% w/v Ionic Krytox surfactant in HFE 7500 (3M) on an 80 × 80 μm drop-making device. Flow rates used are 4000 μL/h for the bead suspension and 12,000 μL/h for the oil. Cell suspension is placed in a 3-mL syringe with a magnetic stir bar and encapsulated in 2% w/v PEGylated surfactant in HFE 7500 on a 80 × 45 μm drop-making device. Flow rates used are 1500 μL/h for the cell suspension and 4000 μL/h for the oil.

### PDM operation for performing linked fluorescence and scRNA-seq analysis in nanoplates

The bead-containing droplets are passed through the PDM device at input rates of 80–120 Hz. Bead-containing droplets are programmably dispensed to each microwell at a maximum printing rate of 3 Hz between nanowells and 10 Hz if printing multiple beads to the same well. Following printing of beads to nanowells, cell-containing droplets are passed through the PDM device at input rates of 80–160 Hz. Cells are dispensed to nanowells at a printing rate of 2–5 Hz. The fluorescence of every cell printed is recorded into a text file along with its nanowell location. Following printing of cells and beads, the nanowell slide is disconnected from its power source, causing droplets to float to the surface, where they are transferred by a P-1000 pipette into a 50-mL conical on ice.

### Sequencing library preparation

The collected emulsions are processed similarly to the Drop-Seq workflow [7]. In brief, the emulsion is broken, beads are collected and reverse transcribed with MMLV reverse transcriptase (Maxima RT, Thermo Fisher), unused primers are degraded with Exonuclease I (New England Biolabs), and beads are washed and PCR amplified. The following modifications are incorporated to account for the low number of beads collected. During the emulsion breakage step, a 0.01% v/v solution of Sarkosyl in 6× SSC is used. During the steps leading up to reverse transcription, a 0.01% v/v Tween-20 solution in 6× SSC is used. Following PCR, the cDNA library is split into two fractions following sequential AmPure bead purification at 0.6× and 2.0× volume ratios as performed the Cite-Seq workflow. Six hundred picograms of cDNA in the fraction containing mRNAs is processed using the Nextera XT kit to form a sequencing library. Five hundred picograms of cDNA in the fraction containing amplified well indexes underwent a second round of PCR to add sequencing adapters. Libraries are pooled and sequenced on an Illumina machine.

### NGS sequencing and optical phenotype data matching

Libraries underwent paired-end sequencing using the custom Drop-Seq primer with a read length of 25 bp for read 1 and 75 bp for read 2. For the mRNA library, reads are processed using the Drop-Seq bioinformatic pipeline. For the well index library, reads are partially processed using the Drop-Seq bioinformatic pipeline, yielding a read-quality filtered and trimmed .sam file with annotations corresponding to UMI and bead barcode positions. The CITE-seq-Count bioinformatic package is used to extract coordinate expression data from the well index sequencing library. Using this coordinate expression matrix, UMI counts for each bead are scaled based on the number of total UMIs on the bead. Next, the off-target noise of each well index is estimated based on the average expression across all beads and subtracted from scaled UMI counts. The top *x*, *y*, and *z* well index captured on each bead is then extracted. Beads which the top well index is not at least three times as abundant as the next most abundant well index for any of the sets of *x*, *y*, and *z* well indexes are removed. The remaining beads are assigned to a nanowell position by matching the most abundant *x*, *y*, and *z* indexes on the bead to a lookup table of the expected *x*, *y*, and *z* positions at each nanowell position. Following position assignment, the bead barcodes of all beads matched at each nanowell position are collected. The columns on the gene expression matrix of all beads matched at the same nanowell position are merged, yielding a revised matrix where the columns represented nanowell positions instead of individual beads. The gene expression matrix is then annotated by recorded cell fluorescence values obtained during printing.

### Gene expression analysis

For the cell cycle experiment, only those cells which expressed at least 300 genes and could be confidently assigned a fluorescence value are processed using the Seurat package in R. Cells are assigned a G2/M and S phase score using Seurat and a list of previous published cell cycle-associated genes [42] which is then used to assign a cell cycle state. Principal component analysis is performed using only the cell cycle-associated genes, and UMAP analysis is then performed on the top 10 principal components. For the PBMC experiment, cells which expressed at least 100 genes and for which there was matching fluorescence data are selected. Gene expression is scaled and normalized using the SCTransform function within Seurat. PCA analysis is performed, and UMAP clustering is performed on the first 6 principal components.

## Supplementary information

**Additional file 1: Movie S1.** Liftoff of drops following removal of power from electrode array.

**Additional file 2: Movie S2.** Printing of four beads into nanowells, slowed 2x.

**Additional file 3.** Supplemental figures and tables.

**Additional file 4.** Python scripts for processing data.

**Additional file 5.** Review history.

## Data Availability

Raw sequencing reads as well as nanowell indexed flow cytometry data are available at the Gene Expression Omnibus under accession GSE136871 [43]. Python scripts for processing sequencing data are available in the supplementary material (Additional file 4).

## References

[1] Zhao X, Guan JL (2011). Focal adhesion kinase and its signaling pathways in cell migration and angiogenesis. Advanced Drug Delivery Reviews.

[2] Giancotti FG (1997). Integrin signaling: specificity and control of cell survival and cell cycle progression. Curr Opin Cell Biol.

[3] Nusse R, Fuerer C, Ching W, Harnish K, Logan C, Zeng A (2008). Wnt signaling and stem cell control.

[4] Hough Shelley R., Laslett Andrew L., Grimmond Sean B., Kolle Gabriel, Pera Martin F. (2009). A Continuum of Cell States Spans Pluripotency and Lineage Commitment in Human Embryonic Stem Cells. PLoS ONE.

[5] Shalek AK, Satija R, Shuga J, Trombetta JJ, Gennert D, Lu D (2014). Single-cell RNA-seq reveals dynamic paracrine control of cellular variation. Nature..

[6] Trapnell C (2015). Defining cell types and states with single-cell genomics. Genome Res.

[7] Macosko EZ, Basu A, Satija R, Nemesh J, Shekhar K, Goldman M (2015). Highly parallel genome-wide expression profiling of individual cells using nanoliter droplets. Cell..

[8] Klein AM, Mazutis L, Akartuna I, Tallapragada N, Veres A, Li V (2015). Droplet barcoding for single-cell transcriptomics applied to embryonic stem cells. Cell..

[9] Fan H. Christina, Fu Glenn K., Fodor Stephen P. A. (2015). Combinatorial labeling of single cells for gene expression cytometry. Science.

[10] Regev A, Teichmann SA, Lander ES, Amit I, Benoist C, Birney E, et al. The human cell atlas. Elife. 2017;6. 10.7554/eLife.27041 PMC5762154 29206104

[11] Gry Marcus, Rimini Rebecca, Strömberg Sara, Asplund Anna, Pontén Fredrik, Uhlén Mathias, Nilsson Peter (2009). Correlations between RNA and protein expression profiles in 23 human cell lines. BMC Genomics.

[12] Angermueller C, Clark SJ, Lee HJ, Macaulay IC, Teng MJ, Hu TX (2016). Parallel single-cell sequencing links transcriptional and epigenetic heterogeneity. Nat Methods.

[13] Penter L, Dietze K, Bullinger L, Westermann J, Rahn HP, Hansmann L (2018). FACS single cell index sorting is highly reliable and determines immune phenotypes of clonally expanded T cells. European Journal of Immunology.

[14] Van Manen HJ, Kraan YM, Roos D, Otto C (2005). Single-cell Raman and fluorescence microscopy reveal the association of lipid bodies with phagosomes in leukocytes. PNAS..

[15] Lane K, Van Valen D, DeFelice MM, Macklin DN, Kudo T (2017). Measuring signaling and RNA-seq in the same cell links gene expression to dynamic patterns of NF-κB activation. Cell Syst.

[16] Wilson NK, Kent DG, Buettner F, Shehata M, Macaulay IC, Calero-Nieto FJ, Castillo MS, Oedekoven CA, Diamanti E, Schulte R, Ponting CP (2015). Combined single-cell functional and gene expression analysis resolves heterogeneity within stem cell populations. Cell Stem Cell.

[17] Kowalczyk MS, Tirosh I, Heckl D, Rao TN, Dixit A, Haas BJ (2015). Single-cell RNA-seq reveals changes in cell cycle and differentiation programs upon aging of hematopoietic stem cells. Genome Res.

[18] Leng N, Chu LF, Barry C, Li Y, Choi J, Li X (2015). Oscope identifies oscillatory genes in unsynchronized single-cell RNA-seq experiments. Nat Methods.

[19] Patel AP, Tirosh I, Trombetta JJ, Shalek AK, Gillespie SM, Wakimoto H (2014). Single-cell RNA-seq highlights intratumoral heterogeneity in primary glioblastoma. Science..

[20] Prakadan SM, Shalek AK, Weitz DA (2017). Scaling by shrinking: empowering single-cell “omics” with microfluidic devices. Nat Rev Genet.

[21] Eng CHL, Lawson M, Zhu Q, Dries R, Koulena N, Takei Y (2019). Transcriptome-scale super-resolved imaging in tissues by RNA seqFISH+. Nature..

[22] Wang Xiao, Allen William E., Wright Matthew A., Sylwestrak Emily L., Samusik Nikolay, Vesuna Sam, Evans Kathryn, Liu Cindy, Ramakrishnan Charu, Liu Jia, Nolan Garry P., Bava Felice-Alessio, Deisseroth Karl (2018). Three-dimensional intact-tissue sequencing of single-cell transcriptional states. Science.

[23] Yuan J, Sheng J, Sims PA (2018). SCOPE-Seq: a scalable technology for linking live cell imaging and single-cell RNA sequencing. Genome Biol.

[24] Yekelchyk M, Guenther S, Preussner J, Braun T. *Mono*- and multi-nucleated ventricular cardiomyocytes constitute a transcriptionally homogenous cell population. Basic Res Cardiol. 2019;114(5):36. 10.1007/s00395-019-0744-z PMC6689038 31399804

[25] Cima Igor, Kong Say Li, Sengupta Debarka, Tan Iain B., Phyo Wai Min, Lee Daniel, Hu Min, Iliescu Ciprian, Alexander Irina, Goh Wei Lin, Rahmani Mehran, Suhaimi Nur-Afidah Mohamed, Vo Jess H., Tai Joyce A., Tan Joanna H., Chua Clarinda, Ten Rachel, Lim Wan Jun, Chew Min Hoe, Hauser Charlotte A. E., van Dam Rob M., Lim Wei-Yen, Prabhakar Shyam, Lim Bing, Koh Poh Koon, Robson Paul, Ying Jackie Y., Hillmer Axel M., Tan Min-Han (2016). Tumor-derived circulating endothelial cell clusters in colorectal cancer. Science Translational Medicine.

[26] Huang H, Sikora MJ, Islam S, Chowdhury RR, Chien Y-H, Scriba TJ (2019). Select sequencing of clonally expanded CD8 + T cells reveals limits to clonal expansion. PNAS..

[27] Nabhan AN, Brownfield DG, Harbury PB, Krasnow MA, Desai TJ (2018). Single-cell Wnt signaling niches maintain stemness of alveolar type 2 cells. Science..

[28] Cole RH, Tang SY, Siltanen CA, Shahi P, Zhang JQ, Poust S (2017). Printed droplet microfluidics for on demand dispensing of picoliter droplets and cells. PNAS..

[29] Siltanen CA, Cole RH, Poust S, Chao L, Tyerman J, Kaufmann-Malaga B, et al. An oil-free picodrop bioassay platform for synthetic biology. Sci Rep. 2018;8(1):1-7. 10.1038/s41598-018-25577-4 PMC5962535 29784937

[30] Fox CB, Nemeth CL, Chevalier RW, Cantlon J, Bogdanoff DB, Hsiao JC (2017). Picoliter-volume inkjet printing into planar microdevice reservoirs for low-waste, high-capacity drug loading. Bioeng Transl Med.

[31] Kiselev VY, Andrews TS, Hemberg M (2019). Challenges in unsupervised clustering of single-cell RNA-seq data. Nat Rev Genet.

[32] McGinnis CS, Murrow LM, Gartner ZJ (2019). DoubletFinder: doublet detection in single-cell RNA sequencing data using artificial nearest neighbors. Cell Syst..

[33] Biočanin M, Bues J, Dainese R, Amstad E, Deplancke B (2019). Simplified Drop-seq workflow with minimized bead loss using a bead capture and processing microfluidic chip. Lab Chip.

[34] Brockhoff G. DNA and proliferation analysis by flow cytometry. In: Cellular diagnostics: basic principles, methods and clinical applications of flow cytometry. S. Karger AG; 2008. p. 390–425.

[35] Becht E, McInnes L, Healy J, Dutertre CA, Kwok IWH, Ng LG (2019). Dimensionality reduction for visualizing single-cell data using UMAP. Nat Biotechnol.

[36] Stansfield BK, Ingram DA. Clinical significance of monocyte heterogeneity. Clin Transl Med. 2015 Dec;4(1):5. 10.1186/s40169-014-0040-3 PMC4384980 25852821

[37] Martinez FO (2009). The transcriptome of human monocyte subsets begins to emerge. J Biol.

[38] Metcalf TU, Wilkinson PA, Cameron MJ, Ghneim K, Chiang C, Wertheimer AM (2017). Human monocyte subsets are transcriptionally and functionally altered in aging in response to pattern recognition receptor agonists. J Immunol.

[39] Cross Sarah E, Jin Yu-Sheng, Tondre Julianne, Wong Roger, Rao JianYu, Gimzewski James K (2008). AFM-based analysis of human metastatic cancer cells. Nanotechnology.

[40] Rubakhin SS, Churchill JD, Greenough WT, Sweedler JV (2006). Profiling signaling peptides in single mammalian cells using mass spectrometry. Anal Chem.

[41] Macaulay IC, Ponting CP, Voet T (2017). Single-cell multiomics: multiple measurements from single cells. Trends Genet.

[42] Nestorowa S, Hamey FK, Pijuan Sala B, Diamanti E, Shepherd M, Laurenti E, Wilson NK, Kent DG, Göttgens B (2016). A single-cell resolution map of mouse hematopoietic stem and progenitor cell differentiation. Blood..

[43] Zhang JQ, Abate AR. Linked optical and gene expression profiling of single cells at high throughput. Datasets. Gene Expression Omnibus. 2019. https://www.ncbi.nlm.nih.gov/geo/query/acc.cgi?acc=GSE136871. Accessed 16 Jan 2020. 10.1186/s13059-020-01958-9 PMC7041248 32093753

