# Supplementary material for: Linked optical and gene expression profiling of single cells at high-throughput
Source: Genome Biol. 2020 Feb 24;21:49. doi: 10.1186/s13059-020-01958-9 (PMC7041248; doi:10.1186/s13059-020-01958-9)
Supplement: Supplementary file 5 — Additional file 5. Review history. [file 13059_2020_1958_MOESM5_ESM.docx]

Review History

**First round of review**

**Reviewer 1**

**Were you able to assess all statistics in the manuscript, including the appropriateness of statistical tests used?**

Yes: The stats looks good

**Were you able to directly test the methods?**

No

**Comments to author:**

Zhang, et al. developed a PDM (printed droplet microfluidics) based platform to jointly profile optical and transcriptome of single cells. The platform includes a printed droplet microfluidic device and an indexed nanowell plate. The microfluidic device enables cell-encapsulating droplets reinjection, acquisition of cell fluorescence signals by optical fibers, sorting droplets containing cells of interest, as well as printing droplets on the nanowells via a printing nozzle. In this process, each cells' fluorescence signals and corresponding nanowell's coordinates were recorded. Since the coordinates of nanowells were uniquely labeled by pre-loaded oligos (coordinate oligos), the optical data were linked to the RNA data after sequencing RNA and coordinate oligos together.

Compared to previously published work, this platform enables co-analysis of single-cell optical and RNA data in a high-throughput manner. For example, the cell fluorescence analysis was achieved at 1 kHz. This nanowells based platform also showed very little cross contamination. Furthermore, the platform was used to study fluorescence and gene expression of DRAQ5 stained Jurkat cells at different cell cycle states. The results showed a general agreement between optical and RNA data when used to determine cell cycle state. This work provides a novel and high-throughput way to study the link between phenotype and genotype at single-cell level. It also brings a new and potential approach to achieve single-cell multi-omics analysis. Nevertheless There are some technical issues that still need to be resolved. I can recommend the publication of this manuscript in Genome Biology after the following issues are addressed.

Specific comments:

1. Compared to the cell analysis at 1 kHz, the throughput of cell/droplet dispensing is only ~5 Hz, which highly limits the potential throughput of this platform. The authors should include discussion about underlying reasons and possible solutions.

2. Page 3, Line 65 mentioned the volume of a nanowell in this work is about ~100 pL. However, the nanowell is clearly far larger than 100 pL as shown in Fig. 2C (judged by the 30-40 μm Drop-seq barcode beads). In addition, add scale bars in Fig. 2C.

3. How did the authors avoid printing droplets containing two cells, since the platform can only detect droplets with or without cells? The authors should compare their work to previous work in the field (e.g. Cao et al., Lab Chip, 2013, 13, 171-178 where multiple cells in a droplet can be detected and examined).

4. In terms of printing efficiency, what is the percentage of nanowells containing both a bead and a cell? What is the recovery rate of printed cells for RNA-seq? As shown in Fig. 3C, it seems that there are many nanowells containing cells but producing no transcriptome data (as black dots in right figure). The authors should clarify.

5. In page 4 line 106, how did the 4-MU dye bind to the beads?

6. In page 5 line 122 and Fig. 3, less than 3000 transcripts per cell (HEK293 or 3T3) are obtained in this work, which is much less than the number of transcripts per cell (44,295 mRNA transcripts from single HEK human cells and 26,044 mRNA transcripts from single 3T3 mouse cells) obtained by Drop-seq. the authors should discuss the factors affecting this results and suggest some potential solutions.

7. The key to link cells' optical signal and dispensing position is to keep the sequence order of droplets after acquiring fluorescence and before printing. Thus, the accuracy of droplet sorting must be 100%. Otherwise, cells' optical data will incorrectly link to RNA data. What is the droplet sorting accuracy for this platform? How did the authors avoid the mis-linking?

**Reviewer 2**

**Were you able to assess all statistics in the manuscript, including the appropriateness of statistical tests used?**

No

**Were you able to directly test the methods?**

No

**Comments to author:**

The Printed Droplet Microfluidics (PDM) technique used here to sort individual cells encapsulated in microfluidic droplets into nanowell plates is very useful and has the potential to be transformative. That said, the technique of sorting cells into nanowell plates was already published by the group in a previous study, as referenced in the manuscript [22,23] and therefore is not novel in itself.

The technique was used in this manuscript to demonstrate that single-cell RNA-seq may be performed on the PDM platform. The authors have demonstrated in the manuscript that it indeed can be. Using a mix of human and mouse cells, the authors also demonstrate that their technique has the necessary single cell specificity. But their claim that this is high-throughput is not well-supported. Current practice in the field does ~ 10,000 cells or more in a typical experiment (Chromium, 10x genomics). The throughput demonstrated in the manuscript is low in that respect (single cell transcripts from 1191 nanowells). This throughput may even be accomplished in the standard 96-well plate format, albeit with tedious pipetting or automated liquid-handling. What PDM technique can do is add (fluorescent) imaging information on top of single cell RNA-seq data, which is definitely a huge advantage. But that is also possible in the commercial platforms (icell8 , Takara Bio) at similar or somewhat higher throughput (~5000 nanowells). I also note that the microfluidic fabrication and instrumentation techniques needed to perform PDM are involved and may not be easy for other labs to adapt.

Jurkat cell-line was used to demonstrate that the system can be used for cell-cycle analysis with single cell resolution. Similar analysis of cell-cycle genes has been performed before and is not new. The cell analysis performed was very basic and there were no new biological insights from the study. With a technique as sophisticated as PDM, so much more could be accomplished by the authors but was not.

**Authors Response**

**Point-by-point responses to the reviewers’ comments:**

Reviewer one

Comment 1: Compared to the cell analysis at 1 kHz, the throughput of cell/droplet dispensing is only ~5 Hz, which highly limits the potential throughput of this platform. The authors should include discussion about underlying reasons and possible solutions.

*Response 1: We thank the Reviewer for bringing up this concern. We anticipate that our platform will have the most impact in applications where a rare cell population needs to be enriched for prior to analysis. If we encapsulate cells at a 1/10 rate in droplets, and the rare cells comprise 5% of all cells, at a 1 kHz cell analysis rate we will print cells at 5 Hz. At that print rate, we can isolate 1000 of those rare cells in just over 3 minutes. We have provided additional data (Figure 5) highlighting this unique advantage of our system and have made changes to the manuscript clarify why this disparity between our cell analysis and printing rates may not be a large issue in specific applications to which our method is best suited.*

Comment 2: Page 3, Line 65 mentioned the volume of a nanowell in this work is about ~100 pL. However, the nanowell is clearly far larger than 100 pL as shown in Fig. 2C (judged by the 30-40 μm Drop-seq barcode beads). In addition, add scale bars in Fig. 2C.

*Response 2: We appreciate the Reviewer bringing to our attention this disparity between the Figures and main text. We clarify that the ~100 pL volume is the typical volume of the microfluidic droplets used in our device and that the nanowells can hold multiple droplets. We have made changes to the text to address this point and have added scale bars to the figure panel in question.*

Comment 3: How did the authors avoid printing droplets containing two cells, since the platform can only detect droplets with or without cells? The authors should compare their work to previous work in the field (e.g. Cao et al., Lab Chip, 2013, 13, 171-178 where multiple cells in a droplet can be detected and examined).

*Response 3: The reviewer raises an excellent point. In droplet scRNA-seq experiments, the two main types of double encapsulations would be homotypic or heterotypic, in which two cells of the same or different type are encapsulated respectively. We agree with the reviewer that doublets of both kinds would add confounding elements to the data analysis, but heterotypic doublets are generally considered more concerning artifacts. However, as we encapsulate cells at a low rate into our droplets, based on Poisson statistics, given a frequency of droplets containing 1 cell to be 10%, those droplets with two or more cells would be less than 1% of the total drop population. In addition, in experiments such as the two cell experiment presented in Figure 3, we can exclude those droplets that have both red and green signal, indicating a heterotypic doublet, and attempt to exclude droplets with abnormally high red or green fluorescence potentially indicative of a homotypic doublet. To reduce the incidence of homotypic doublets, we can further dilute cells during droplet encapsulation such that a cell is present in only 5% or 3% of drops, although this would reduce the print speed. The ability to sort only desired cells during the array loading process is a unique aspect of our method. We have amended our discussion of Figure 3 in response to this comment.*

Comment 4: In terms of printing efficiency, what is the percentage of nanowells containing both a bead and a cell? What is the recovery rate of printed cells for RNA-seq? As shown in Fig. 3C, it seems that there are many nanowells containing cells but producing no transcriptome data (as black dots in right figure). The authors should clarify.

*Response 4: We thank the reviewer for bringing up this point. We agree that recovery rate is an important parameter and should be clarified in the manuscript. Because we print beads first to each well, then add cells, we can consider the printing efficiency to be the product of the bead printing efficiency and the cell printing efficiency. From Figure 2C, we observed that all wells contained beads; from Figure 3C, 16/2352 wells do not contain any linked optical data, which consider as missed cell printing events. Taken together, we expect that 99.3% of wells contain bead and cells at the time of printing.*

*We define recovery rate as the rate at which we recover quality single cell transcriptomes. In Figure 3, this figure is 1204/2352 wells, or 51.2%. There are several main contributing factors that contribute to this number being lower than the printing efficiency:*

*1. Following printing, some droplets, especially near the edges of the array, are never suctioned up into the collection tube.*

*2. During library prep, one performs a series of washes between steps. With each wash, a small fraction of beads is lost from the pellet, leading to cases where all the beads originating from a given well are lost during library prep.*

*3. Some beads are quality filtered during bioinformatic analysis.*

*Due to these factors, the recovery rate may be lower than the true cell recovery rate. The reviewer raises an excellent point, and we have amended the manuscript text to include more discussion about cell recovery rate.*

Comment 5: In page 4 line 106, how did the 4-MU dye bind to the beads?

*Response 5: We appreciate the reviewer bringing up this question. We believe that the 4-MU dye binds by a noncovalent interaction with the Drop-Seq beads. The Drop-Seq beads are made of an acrylate polymer, which can interact with 4-MU. As 4-MU is insoluble in water, it remains partitioned within the beads following trapping within the beads for the duration of the experiment. As we believe that this a question that others may have, we have amended the manuscript to include this hypothesized 4-MU binding mechanism.*

Comment 6: In page 5 line 122 and Fig. 3, less than 3000 transcripts per cell (HEK293 or 3T3) are obtained in this work, which is much less than the number of transcripts per cell (44,295 mRNA transcripts from single HEK human cells and 26,044 mRNA transcripts from single 3T3 mouse cells) obtained by Drop-seq. the authors should discuss the factors affecting this results and suggest some potential solutions.

*Response 6: We agree with the reviewer that this interpretation of our sequencing results may raise concern, as RNA capture efficiency is an important parameter for scRNA-seq methods. We are using the same capture chemistry as in the Drop-Seq workflow, so we expect to receive similar capture efficiencies. The data presented in Figure 3 was sparsely sequenced; in an earlier experiment we sequenced fewer cells and thus were able to sequence deeper. On average, we obtained 17500 and 15200 transcripts from mouse and human cells. We and others have observed batch to batch performance variability with the beads, which may also influence disparities in transcript capture efficiency. We summarize these results in Supplemental Figure 1 and have modified the manuscript to include discussion of the points raised by the reviewer.*

Comment 7: The key to link cells' optical signal and dispensing position is to keep the sequence order of droplets after acquiring fluorescence and before printing. Thus, the accuracy of droplet sorting must be 100%. Otherwise, cells' optical data will incorrectly link to RNA data. What is the droplet sorting accuracy for this platform? How did the authors avoid the mis-linking?

*Response 7: The reviewer raises an excellent point. We have previously demonstrated near-perfect accuracy when attempting to sort a pre-defined pattern of cells or drops (Cole, PNAS, 2017). Indeed, except for rare cases where the printer fails to sort an event, we obtained linked fluorescence data for 99.3% of droplets, as shown in Figure 3C. We observed that 0.6% of wells contained the incorrect cell type and 1.8% of wells contained doublets. The former is highly likely to have arisen from sorting errors while sorting errors partially contribute to the latter. We appreciate the commentary provided by the reviewer and have amended the manuscript to provide more discussion about these points.*

Reviewer Two

Comment

[The author’s] claim that this is high-throughput is not well-supported. Current practice in the field does ~ 10,000 cells or more in a typical experiment (Chromium, 10x genomics). The throughput demonstrated in the manuscript is low in that respect (single cell transcripts from 1191 nanowells). But that is also possible in the commercial platforms (icell8 , Takara Bio) at similar or somewhat higher throughput (~5000 nanowells). The cell analysis performed was very basic and there were no new biological insights from the study. With a technique as sophisticated as PDM, so much more could be accomplished by the authors but was not.

*Response*

*We thank Reviewer 2 for their insight and providing input on our manuscript. We believe that our platform should be compared to commercially available platforms that also have the capability to integrate fluorescence measurements with RNA-seq data. To our knowledge, the main platforms in this space include the Fluidigm integrated fluidic circuit (IFC) and the Takara Bio iCell8. The former is lower throughput, processing 96 cells per experiment. The iCell8 platform contains a 5184 nanowell chip, but cells must be deposited stochastically into the wells following a Poisson distribution. If we follow Poisson statistics and maximize the number of wells in which there is one cell, about 36.8% of wells will contain a cell, meaning that the iCell8 can process about 1900 cells per experiment. However, in cases with a rare cell type of interest present at 5% abundance or less, at most 95 of those cells will be processed. Researchers have now identified rare cells which can tune the pathogenesis of cancer, heal damaged tissue, and modulate immunological memory. To further study cells such as these using single cell sequencing, we need a technique to isolate rare cells prior to performing the sequencing library prep.*

*The fluorescence phenotyping capabilities of PDM also allow us to enrich for rare cell types during the loading of the array. We have performed an additional experiment demonstrating our ability to enrich for specific cell types from peripheral blood mononuclear cells (PBMCs). Phenotyping of PBMCs is routinely done in clinical and research settings; for example, in Acute Myeloid Leukemia (AML), cancerous blast cells may constitute less than 5% of all leukocytes but are the cells of greatest interest. Using fluorophore conjugated antibodies, we used PDM to enrich for CD14+ and CD16+ cells from a human PBMC sample. Prior to microfluidic cell sorting, we counted the number of CD16+ cells in our sample and estimated that they constituted only 3% of all cells, and about 8x less abundant than CD14+ cells. We show in Figure 5B that we are able to recover gene expression phenotypes matching those found in CD16+ PBMCs and also show that if we limit subsample the CD16+ analyzed such that their numbers equal 1/8 of the CD14+ cells analyzed, we lose the ability to resolve the CD16+ phenotypes by RNA-seq. This comment by Reviewer 2 is excellent and has pointed out a major issue in demonstrating the innovation of our method. We have taken the time to collect additional data to demonstrate this important innovation of our method, unique compared to similar platforms that are commercially available.*

**Second round of review**

**Reviewer 1**

The revision is satisfactory. I can recommend acceptance.
